# Enhanced YOLOv11 framework for high precision defect detection in printed circuit boards

**DOI:** 10.1038/s41598-025-27415-w

**Published:** 2025-11-26

**Authors:** Zeinab F. Elsharkawy

**Affiliations:** Engineering Department, Nuclear Research Center, Egyptian Atomic Energy Authority (EAEA), Cairo, Egypt

**Keywords:** Deep learning, Printed circuit boards, YOLOv11, Attention mechanisms, EIoU, CARAFE, EMA, Electrical and electronic engineering, Computer science

## Abstract

This paper presents YOLOv11-PCB, an enhanced deep learning framework specifically designed for automated defect detection in Printed Circuit Boards (PCBs). PCBs are fundamental components in modern electronics, and their reliability hinges on precise defect localization. Conventional inspection methods, such as manual inspection and traditional image processing, are limited by subjectivity, high labor intensity, and poor generalization across diverse PCB layouts. To address these challenges, we propose YOLOv11-PCB. It integrates three key innovations: (1) an Efficient Multi-Scale Attention (EMA) module for adaptive feature extraction, (2) a Content-Aware ReAssembly of Features (CARAFE) mechanism for dynamic receptive field adjustment, and (3) a refined Efficient Intersection over Union (EIoU) loss function that optimizes bounding box regression. Extensive experiments conducted on two benchmark PCB defect datasets validate the effectiveness of our proposed approach. YOLOv11-PCB achieves a mean average precision of 99.5% (mAP@0.5) and 90.7% (mAP@0.5:0.95) on the Peking University PCB dataset, reflecting a 9.7% improvement over the baseline YOLOv11. On the DeepPCB dataset, it reaches 98.9% and 81%, respectively, showing notable gains, including a 1.8% improvement over the baseline. The system maintains real-time processing capabilities at 227.2 frames per second (FPS), outperforming state-of-the-art methods in both detection accuracy and computational efficiency. These results highlight YOLOv11-PCB’s robustness in identifying critical PCB defects, including solder bridges, missing components, and micro-scale fractures, while meeting the stringent throughput requirements of industrial production lines.

## Introduction

As the foundation for mechanical and electrical connections in electronic devices, PCBs are essential to contemporary electronics^1–3^. Advancements in electronic technology have surged the demand for high-performance, reliable PCBs. However, the complexity of PCB manufacturing introduces various defects—such as missing holes, open circuits, mouse bites, spurious copper, and short circuits—which can compromise functionality, safety, and product longevity. Thus, developing efficient and precise defect detection and localization methods is critical to maintaining quality standards, reducing production costs, and preventing system failures^4^.

Conventional PCB defect detection techniques, including manual inspection, functional testing, and traditional image processing, suffer from notable limitations. Manual inspection is labor-intensive, subjective, and susceptible to human error due to fatigue and inconsistent defect visibility. Functional testing, though effective for electrical fault detection, fails to identify visual or structural anomalies and often incurs high costs and lengthy testing cycles. Traditional image processing methods—such as template matching, SIFT, SURF^5^, and morphological operations—have notable limitations. They struggle to generalize across diverse PCB layouts, complex textures, and variations in lighting, orientation, and defect morphology. These approaches also face stringent operational constraints, limiting their robustness and real-time applicability in industrial settings^6,7^.

Deep learning has emerged as a transformative solution for PCB defect detection, offering superior generalization, accuracy, and nondestructive inspection capabilities. Architectures like Faster R-CNN^1,8^, SSD^9^, and various YOLO variants^4,6,10–22^ have demonstrated promising results. Recent studies have explored YOLOv11’s architectural enhancements, such as improved feature extraction and anchor-free detection heads^23–25^. Despite these advances, YOLOv11 struggles with detecting minute PCB defects due to limited spatial resolution and insufficient attention to fine-grained features. To overcome these limitations, we integrate EMA, CARAFE, and EIoU into YOLOv11.

In recent studies, attention processes have been gradually included into computer vision tasks to enhance the performance of deep learning models^26,27^. By dynamically concentrating resources on the most noticeable and discriminative features, these strategies maximize computing economy while improving robustness and accuracy. Despite the growing adoption of deep learning in PCB inspection, existing models often struggle with:

1. Detection of minute and subtle defects, particularly those occupying less than 2% of the image area.
2. Maintenance of real-time processing speeds to meet high-throughput production demands.
3. Deployment of lightweight models suitable for resource-constrained edge devices^4,14,16,21–23^.

Attention mechanisms and advanced upsampling techniques have been proposed individually. However, few frameworks combine these strengths in a unified and computationally efficient manner. To bridge this gap and build upon these advancements, we introduce YOLOv11-PCB, an improved framework that combines three well-proven components—EMA, CARAFE, and EIoU—into a unified YOLOv11-PCB architecture designed specifically for PCB inspection. The EMA module is utilized for adaptive feature extraction across a range of defect sizes^26,28,29^, the CARAFE mechanism is utilized for dynamic receptive field adjustment^15,22,27^, and the EIoU loss function is utilized for accurate bounding box regression^30–32^. While EMA, CARAFE, and EIoU are each well-established in existing research, their combined implementation within a unified, lightweight, and high-speed YOLOv11-based framework tailored for PCB defect detection marks a novel engineering advancement. Our results demonstrate that this combination yields significant performance gains in both accuracy and inference speed, validating the effectiveness of this tailored design.

To comprehensively evaluate the effectiveness and generalizability of the proposed YOLOv11-PCB model, two benchmark datasets were utilized: the Peking University PCB dataset^33^ and the DeepPCB dataset^34^. The Peking University dataset comprises 21,336 high-resolution color images representing six common PCB defect types, while the DeepPCB dataset contains 3,600 grayscale images captured under real-world industrial conditions. This dual-dataset strategy enables robust validation across diverse imaging modalities and defect complexities. Experimental results demonstrate that YOLOv11-PCB delivers outstanding performance on both datasets, achieving an mAP@0.5 of 99.5% and mAP@0.5:0.95 of 90.7% on the Peking University dataset and 98.9% mAP@0.5 and 81% mAP@0.5:0.95 on the DeepPCB dataset. These results significantly outperform existing state-of-the-art models while maintaining real-time processing speeds of 227.2 FPS.

To bridge the precision-speed gap in PCB defect detection, we propose a novel integration of three powerful components—EMA, CARAFE, and EIoU loss—within the YOLOv11 architecture. To the best of our knowledge, this is the first application of this combined architecture for PCB inspection tasks. Each of these modules has been explored individually in other contexts. However, their unified integration into a lightweight, real-time object detection framework—specifically optimized for high-resolution, small-defect PCB characteristics—represents a distinctive engineering contribution. The cumulative and synergistic benefits of these modules are substantiated through detailed ablation studies, which reveal measurable improvements in both detection accuracy and inference speed. This design strikes a practical balance between high-precision localization and real-time processing—key requirements for industrial deployment.

The key contributions include

1. A novel YOLOv11-based architecture optimized for PCB defect detection.
2. Integration of EMA and CARAFE for superior multi-scale feature representation.
3. An EIoU loss function specifically tailored for small-defect localization.
4. Comprehensive validation was performed using an augmented Peking University dataset and the DeepPCB dataset.

The following is how the rest of the paper is organized: Sect. 2 reviews related work in PCB defect detection and deep learning-based object detection. Section 3 details our proposed methodology, including network architecture and loss function. Section 4 presents experimental results and analysis. Finally, Sect. 5 concludes the paper and outlines future research directions.

## Literature review

Ensuring the reliability and performance of electronic devices hinges on effective PCB defect detection. Conventional inspection techniques, such as manual inspection, electrical testing, and Automatic Optical Inspection (AOI), suffer from several drawbacks, including subjectivity, high equipment dependency, and susceptibility to environmental variations^1–3^. In recent years, machine vision-based approaches—encompassing image processing, machine learning, and deep learning—have gained prominence. While traditional image processing methods often struggle with accuracy, deep learning techniques, particularly Convolutional Neural Networks (CNNs), have verified superior performance due to their high precision, adaptability to variations, automated feature extraction, and end-to-end learning capabilities^5,35–41^.

Various deep learning architectures have been investigated for PCB defect detection. Two-stage object detectors, such as Faster R-CNN, initially generate regions of interest before classification. Enhancements like the Selective Feature Attention-Pixel Shuffle Pyramid (SF-PSPyramid) have been introduced to improve the detection of minute defects^35^. Chang et al.^5^ proposed a hybrid approach combining Particle Swarm Optimization (PSO) threshold segmentation with Speeded-Up Robust Features (SURF) for defect identification. Meanwhile, Zhao et al.^40^ developed the Adaptive Key Point Localization Network (AKPLNet), which integrates a residual pyramid heat mapping network, an adaptive tree-structured region proposal network, and a key point regression algorithm to achieve precise defect localization. This model attained mAP scores of 96.9% and 99.0% on the PCB-Master and DeepPCB-Master datasets, respectively. Wu et al.^42^ introduced an end-to-end Efficient Model (EEMNet) tailored for detecting tiny surface defects, achieving an accuracy of 99.1% at 77 frames per second (FPS), underscoring the adaptability of deep learning frameworks to varying defect sizes.

Single-stage detectors, such as YOLO (You Only Look Once), offer faster inference speeds by predicting object classes and locations in a single step. Several YOLO variants—including YOLOv3^4,43^, YOLOv4^2^, YOLOv5^4,43–45^, YOLOv7^4,8,44^, YOLOv8^2,4,10,14,43,44^, and YOLOv9^4^—have been optimized to address challenges specific to PCB defect detection, such as small defect sizes, complex backgrounds, and real-time processing demands. For instance, Weifeng^10^ achieved an F1-Score of 98% and a mAP@0.5 in PCB defect localization, indicating the effectiveness of YOLO-based approaches.

Recent advancements in YOLO-based models have further enhanced PCB defect detection. Du et al.^6^ incorporated Mobile Inverted Residual Bottleneck (MBConv) modules, Convolutional Block Attention Modules (CBAM), and Bidirectional Feature Pyramid Networks (BiFPN) into YOLOv5, achieving an mAP@0.5 of 99.0% at 69.5 FPS. Another study^4^ replaced YOLOv5’s backbone with FasterNet, attaining a precision of 98.0%, a recall of 98.9%, an mAP@0.5 of 98.8%, and an inference speed of 104.8 FPS with 11.5 Giga Floating point operations per second (GFLOPs). Tang et al.^13^ introduced PCB-YOLO, an optimized variant of YOLOv5 incorporating K-means + + anchor box clustering, Swin Transformer blocks, and depth-wise separable convolutions, realizing 95.97% mAP@0.5 at 92.5 FPS. Zhou et al.^46^ proposed MSD-YOLOv5, which combines MobileNet-v3 with CSPDarknet53 to balance accuracy, speed, and model compactness. Patel^47^ leveraged a Multi-level Spatial Attention Pyramid Generative Adversarial Network (MuSAP-GAN) to enhance defect detection robustness, achieving 95.24% accuracy. Xiao et al.^48^ developed CDI-YOLO, integrating a coordinate attention mechanism and an optimized loss function with YOLOv7, reaching 98.3% mAP@0.5 at 128 FPS. Additionally, Ghost-YOLOv8^2^ employed Ghost Convolution and Wise-IoU (WIoU) loss to improve real-time performance, achieving 125 FPS while increasing mAP@0.5 by 0.8%.

Recently, YOLOv11 has emerged as a powerful object detection framework, offering improvements in inference speed and detection accuracy through architectural refinements such as enhanced feature extraction and anchor-free detection heads^23–25^. While YOLOv11 provides a strong baseline for general object detection tasks, it still faces challenges when applied to PCB defect detection. Specifically, its performance degrades when detecting minute defects that occupy a small fraction of the image area, and it lacks specialized mechanisms for preserving fine-grained spatial features critical in PCB inspection. These limitations motivate the enhancements proposed in this work, including the integration of EMA for multi-scale attention, CARAFE for content-aware upsampling, and EIoU for improved bounding box regression.

## The proposed approach

### YOLOv11-PCB architecture

**Fig. 1:**
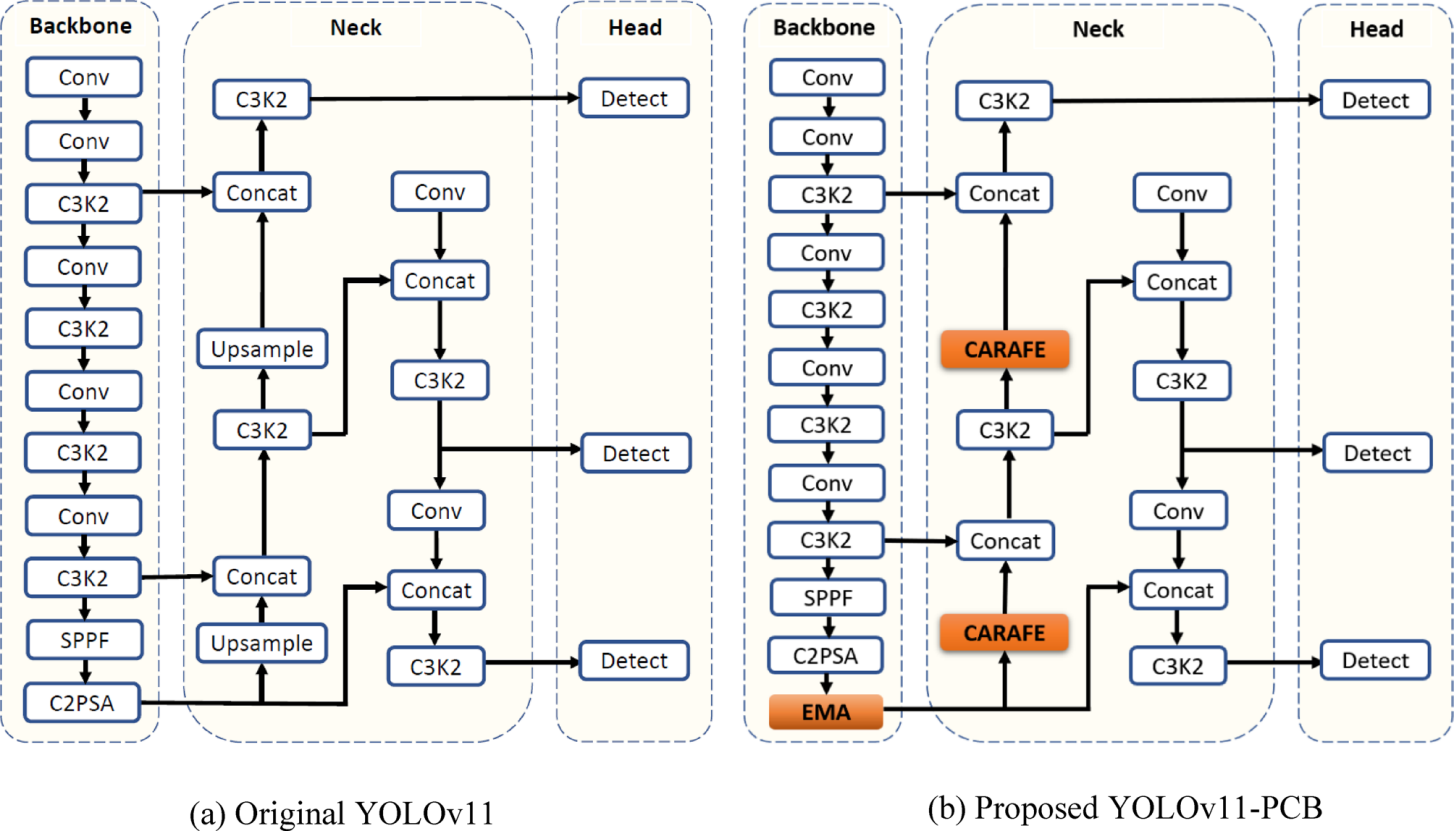
The original and adapted YOLOv11 structures, highlighting the integration of EMA and CARAFE modules.

The proposed YOLOv11-PCB framework builds upon the original YOLOv11 architecture, which features a modular design comprising a backbone for feature extraction, a neck for feature fusion, and a head for detection. We retain the original three-stage structure of YOLOv11. However, we introduce key modifications aimed at (1) enhancing feature representation for small defects, (2) preserving spatial detail during upsampling, and (3) improving localization accuracy. Specifically, we integrate an EMA module into the backbone to improve feature representation across scales. In the neck, we substitute traditional upsampling with the CARAFE module to preserve spatial detail during resolution recovery. Finally, we replace the standard IoU loss in the detection head with the EIoU loss function to improve bounding box regression, particularly for small and irregular defects. These enhancements are illustrated in Fig. 1 and detailed in the following subsections.

The YOLOv11-PCB framework introduces a novel deep learning architecture optimized for high-precision defect localization in PCBs. As represented in Fig. 1, the model contains three principal components—Backbone, Neck, and Head—each enhanced with advanced mechanisms to improve computational efficiency and detection accuracy for industrial inspection tasks.

#### Backbone: multiscale feature extraction

The backbone is designed to extract multi-scale features with minimal computational overhead:

- Conv Layers: Standard convolutional layers for initial feature extraction.
- C3K2 Blocks: Lightweight CSP-based bottlenecks with a 2 × 2 kernel size, reducing parameters while preserving feature richness. These replace traditional C3 blocks for faster inference.
- SPPF (Spatial Pyramid Pooling Fast): Aggregates multi-scale context efficiently, improving detection of defects at varying sizes (e.g., solder bridges vs. micro-cracks).
- C2PSA (Cross-Stage Partial with Spatial Attention): Enhances focus on defect regions by combining CSP with spatial attention, critical for small or occluded anomalies.
- EMA: The Efficient Multi-Scale Attention module enhances feature fusion and multi-scale representation. For a detailed explanation, please refer to Sect. “2.2 EMA”.

The repeated use of C3K2 (instead of larger kernels) and EMA reduces GFLOPs while maintaining accuracy, crucial for real-time PCB inspection.

#### Neck: feature fusion and refinement

The Neck refines and merges Backbone features through a hybrid pathway:

- C3K2 Blocks: Maintain consistency with the Backbone’s lightweight design.
- Concat + CARAFE: This content-aware upsampling mechanism improves spatial resolution during feature reassembly. See Section “2.3 CARAFE” for full architectural details.
- Bi-directional Flow: Features are concatenated and upsampled iteratively (e.g., Concat → CARAFE → C3K2), enabling precise defect localization.

**Fig. 2:**
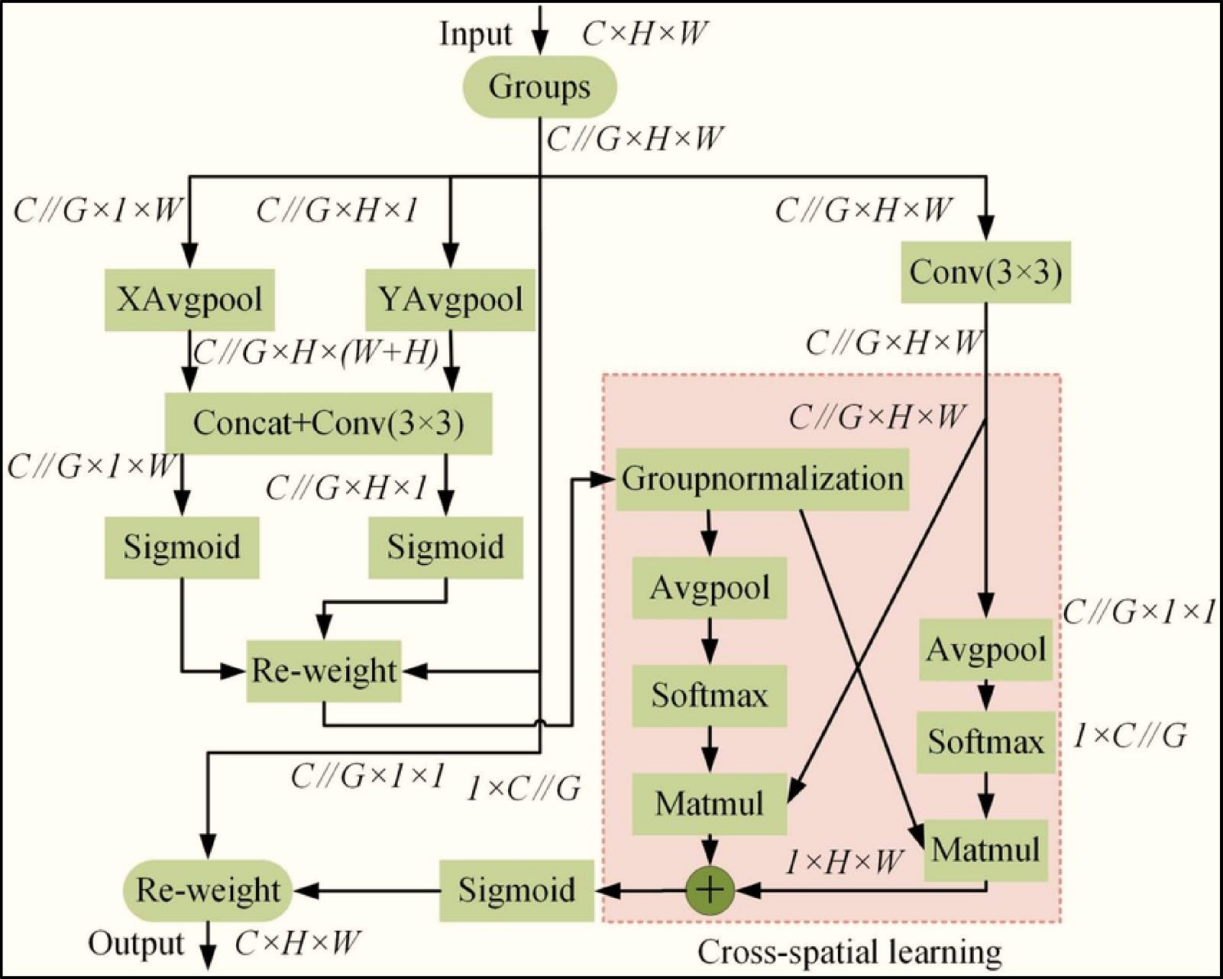
Architecture of the efficient multi-scale attention (EMA) module, showing excitation and modulation stages for multi-scale feature refinement^29^.

CARAFE’s content-aware upsampling outperforms fixed-kernel methods (e.g., bilinear interpolation) for irregular PCB defects like hairline cracks or misaligned pads.

#### Head: detection and localization

The head is streamlined for efficiency:

- Single Detect Layer: Employs an anchor-free approach (similar to YOLOv8), which predicts object locations directly without relying on predefined anchor boxes, thereby reducing complexity.
- Task-Specific Outputs: Directly predicts bounding boxes, masks, and class scores without redundant branches, accelerating inference.

**Fig. 3:**
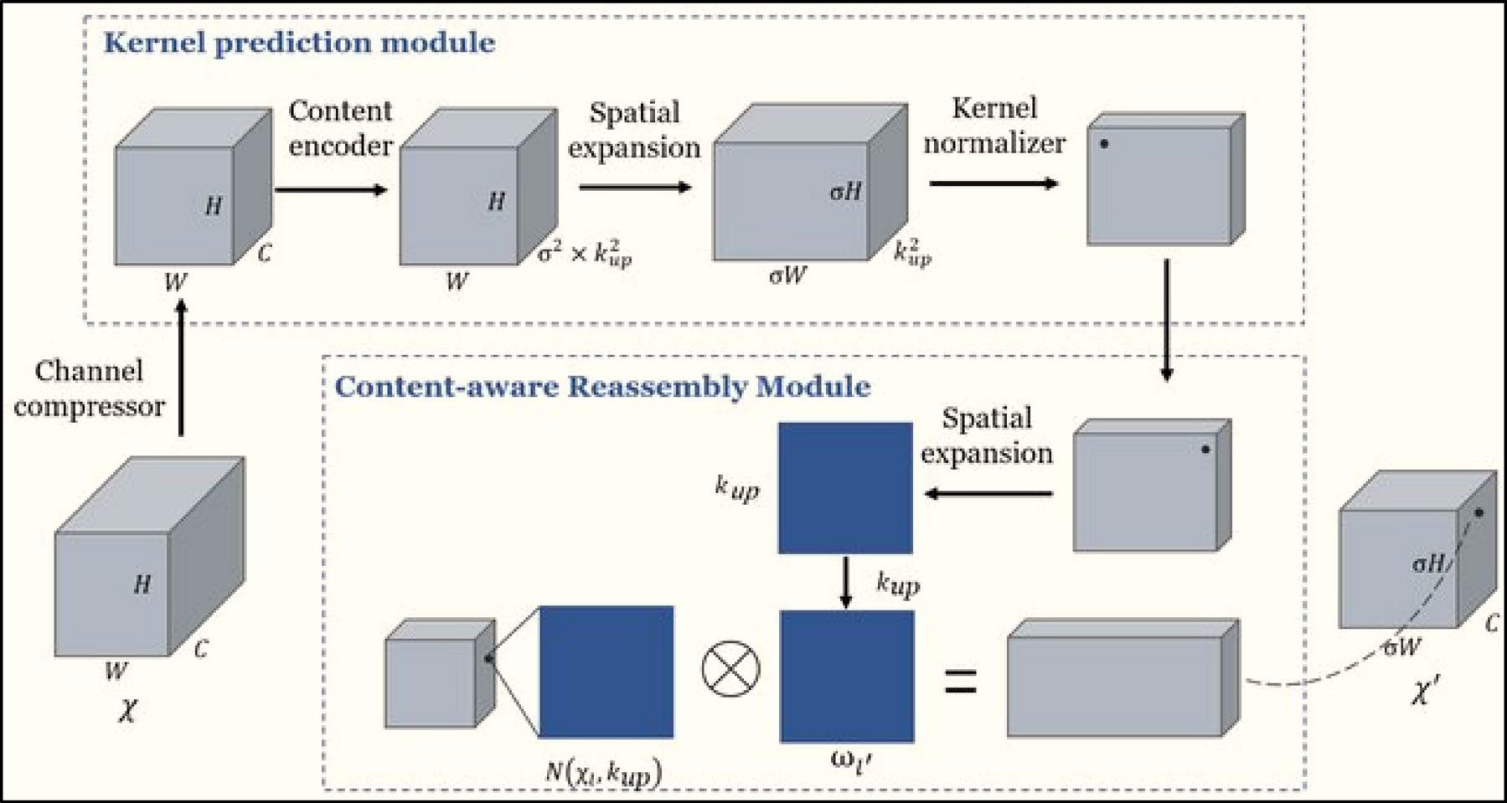
Structure of the Content-Aware ReAssembly of FEatures (CARAFE) attention mechanism module^22^.

This minimalist design reduces latency, making the model suitable for high-speed production-line inspection. The integration of EMA (backbone) and CARAFE (neck) enables YOLOv11-PCB to dynamically adjust its receptive field, capturing multi-scale defect features with superior accuracy. Collectively, these innovations position YOLOv11-PCB as a modern solution for real-time PCB inspection, where precision, scalability, and computational efficiency are critical.

Integrating EIoU loss into YOLOv11’s network structure significantly enhances localization accuracy for PCB defect detection. Unlike traditional IoU or CIoU losses, the EIoU loss function improves bounding box regression, especially for small and irregular defects. A full explanation is provided in Section “2.4 EIoU.”

### EMA

The EMA module improves feature representation by restructuring channel dimensions into batch-wise sub-features, ensuring balanced spatial semantic distribution across groups^26,28,29^. Unlike traditional methods, EMA models fine-grained pixel-level associations through cross-dimensional interactions. It also recalibrates channel weights within parallel branches using global contextual information. As illustrated in Fig. 2, the EMA module processes input features through excitation and modulation stages. The excitation stage computes a similarity matrix to assess feature relevance, while the modulation stage applies softmax-normalized weights to enhance task-relevant features. This structure enables the model to focus on subtle defect patterns across multiple scales. At its core, EMA extends traditional attention mechanisms by integrating excitation and modulation processes:

- The excitation phase evaluates feature relevance by computing a similarity matrix (via inner products) between input features and learnable parameters. Each matrix element quantifies the alignment between a local feature and the task objective, with higher values indicating greater importance.
- The modulation phase dynamically adjusts feature weights based on this matrix, typically employing softmax normalization to derive a probabilistic weighting vector. This vector is then used to compute a contextually weighted feature representation through linear combination.

By selectively amplifying task-relevant features while suppressing noise, EMA enhances model discriminability. We integrate this mechanism to improve detection accuracy through more robust feature learning.

### CARAFE

The CARAFE module is an advanced feature upsampling mechanism that dynamically adapts to local content, offering superior performance over traditional interpolation methods (e.g., bilinear or transposed convolution)^15,22,27^. Figure 3 visualizes the CARAFE module’s four-stage process: channel reduction, adaptive kernel generation, kernel normalization, and contextual feature assembly. This design allows the model to reconstruct high-resolution features with greater spatial accuracy, which is essential for detecting fine-grained PCB anomalies. Its operation can be conceptually decomposed into four sequential stages:

1. Channel Reduction: A 1 × 1 convolution is used to compress the input feature map, which has dimensions of *H*×*W*×*C*. This reduces the depth of the map to $\:{C}_{m}$ (the number of compressed channels).
2. Adaptive Kernel Generation: The compressed features are processed by a Kencoder×Kencoder *(3 × 3)* convolutional layer to predict position-specific upsampling kernels. These kernels, each of size $\:\text{K}\text{u}\text{p}\times\:\text{K}\text{u}\text{p}\:$*(5 × 5)*, are generated with spatial dimensions expanded by the scaling factor ‘s’ $\left( {sH~ \times ~sW~ \times ~Kup^{2} } \right)$.
3. Kernel Normalization: Each predicted kernel undergoes softmax normalization across its spatial dimensions, ensuring the kernel weights sum to unity. This normalization maintains the energy-preserving property of the feature transformation.
4. Contextual Feature Assembly: The final upsampled features are produced through a content-aware convolution operation, where the normalized kernels perform weighted aggregation of local features from the original input. This adaptive recombination preserves structural information while achieving the desired spatial resolution enhancement.

CARAFE strikes a balance between computational cost and upsampling quality, making it a popular choice for modern architecture.

### EIoU

Efficient IoU is an advanced solution for bounding box regression^30–32^. It is a major progress in IoU-based loss functions, especially made to improve regression performance in object detection while getting beyond the drawbacks of conventional IoU, GIoU, DIoU, and CIoU losses. Unlike traditional IoU, which suffers from gradient vanishing when boxes don’t overlap, EIoU ensures stable optimization by decomposing the regression error into three key components:

- Overlap Loss $\:(1-IoU)$ Maintains the fundamental intersection measure.
- Center Distance Loss $\left( {\rho ^{2} \left( {b,b_{{gt}} } \right)/c^{2} } \right)$ Penalizes misalignment between predicted and ground truth box centers, improving localization accuracy.
- Dimensional Loss $\rho ^{2} \left( {w,w_{{gt}} } \right)/C_{w}^{2} ~ + ~\rho ^{2} \left( {h,h_{{gt}} } \right)/C_{h}^{2} )~$– Explicitly minimizes width and height discrepancies, unlike CIoU’s flawed aspect ratio term, which only considers proportional differences and creates conflicting gradients.

The complete EIoU formulation:

$$\:{L}_{EIoU}=1-IoU+\:\:\:\frac{{\rho\:}^{2}\left(b,{b}_{gt}\right)}{{c}^{2}}+\:\:\frac{{\rho\:}^{2}\left(w,{w}_{gt}\right)}{{C}_{w}^{2}}+\:\frac{{\rho\:}^{2}\left(h,{h}_{gt}\right)}{{C}_{h}^{2}}$$

Where:

- $\:b=\:(\text{x},\text{y},\text{w},\text{h})\:$ is the predicted bounding box.
- $\:{b}_{gt}=\:({\text{x}}_{\text{g}\text{t}},{\text{y}}_{\text{g}\text{t}},{\text{w}}_{\text{g}\text{t}},{\text{h}}_{\text{g}\text{t}})\:$ is the ground truth.
- $\rho ^{2}$ denotes squared Euclidean distance.
- $\:c$ is the diagonal length of the smallest enclosing box.
- $\:{C}_{w}\:$and $\:{C}_{h}$ are the width and height of the smallest enclosing box.
- $\:IoU$ is the intersection over union between the predicted and ground truth bounding boxes.

This formulation ensures stable gradients even when boxes do not overlap and provides more precise localization by explicitly penalizing center misalignment and size discrepancies.

Unlike CIoU’s problematic aspect ratio term, which creates conflicting gradients and ignores scale differences—EIoU directly minimizes dimensional discrepancies while maintaining stable gradients, even for non-overlapping boxes. This formulation enables faster convergence, more precise localization, and better handling of varying object scales, making EIoU superior for modern object detectors. By combining robust overlap measurement with independent size and center penalties, EIoU overcomes the regression ambiguities of earlier approaches, establishing itself as a more effective loss function for accurate bounding box prediction.

## Experiments

### Experimental environment

The proposed model was implemented and evaluated on a high-performance computing system with the following specifications:

Hardware and Software Configuration:

- Processor: AMD Ryzen Threadripper 3960 × (24 cores @ 3.79 GHz base clock).
- GPU: Dual NVIDIA GeForce RTX 3070 (8GB GDDR6 each) in parallel configuration.
- System Memory: 32GB DDR4 RAM.
- Operating System: Windows 11 Pro.
- Python 3.10.
- Pytorch framework 2.4.1.

A batch size of 16, an epoch of 300, and an image size of 608 are employed during training. We used the Adam optimizer for network optimization. Furthermore, we employed a learning rate of 0.001, a momentum of 0.900, and a weight decay of 0.0005 to avoid overfitting during training.

### Experimental results and analysis of Peking university database

**Fig. 4:**
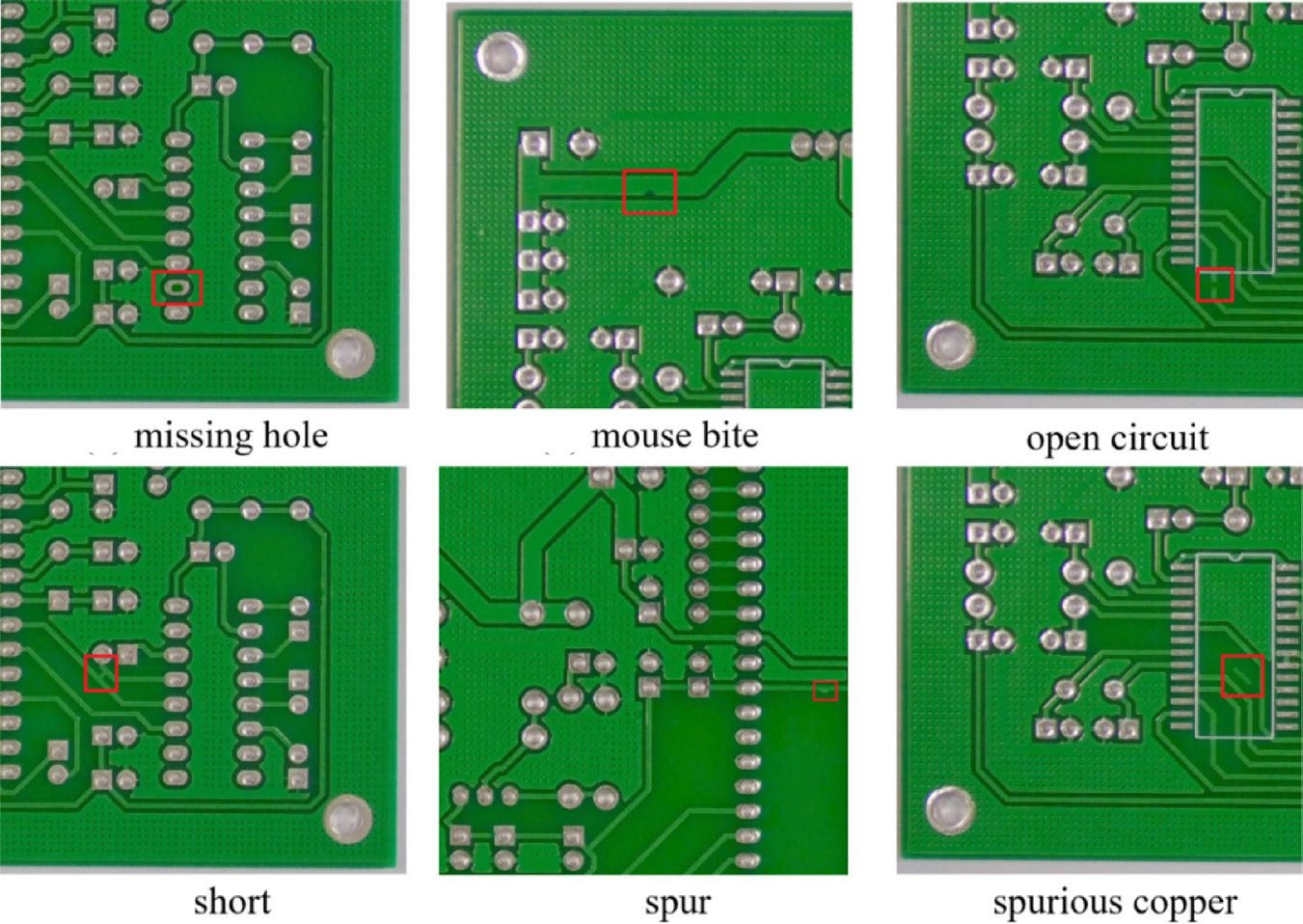
Experimental data covering six frequently occurring PCB surface flaw categories.

**Fig. 5:**
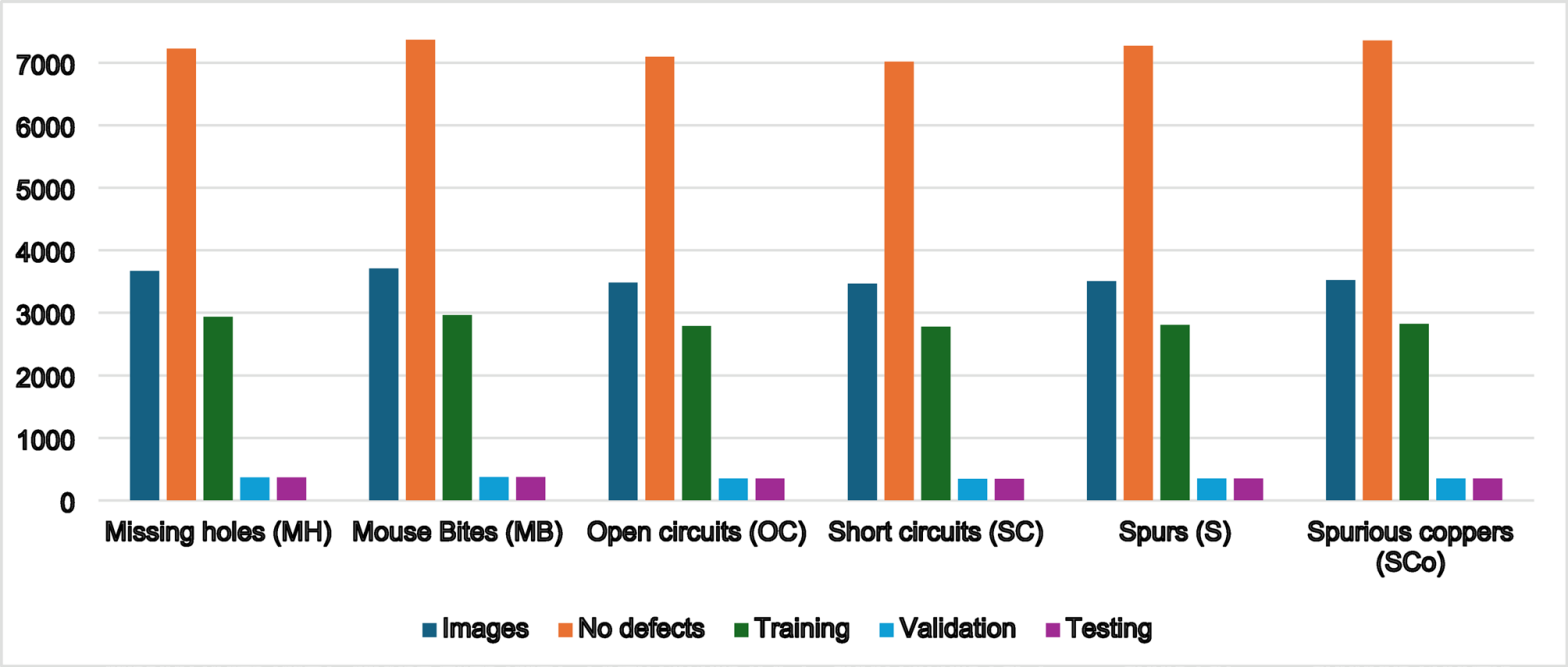
Details of the Peking University PCB defect dataset.

For this investigation, we used Peking University’s Intelligent Robot Open Laboratory’s PCB Defect Dataset, which is openly accessible, for this investigation^33^. The original dataset comprises 10,668 high-resolution images of six common PCB manufacturing defects, including missing holes, short circuits, and spurious copper patterns. To enhance diversity and generalization, we applied data augmentation (multi-angle rotations, Gaussian noise, and color space perturbations). This augmentation strategy effectively doubled our dataset size to 21,336 samples while maintaining balanced class distribution. As shown in Fig. 4, the six defect categories exhibit varying degrees of visual salience, with some defects occupying less than 2% of the total image area. We divided the augmented dataset into three subsets: 80% for training, 10% for validation, and 10% for testing. This ensured no data leakage between sets. Complete statistical breakdowns of defect distributions and dataset characteristics are provided in Table 1; Fig. 5.

The detection challenge is compounded by several factors:

1. Sub-millimeter defect sizes requiring high-resolution analysis.
2. Low-contrast visual signatures often indistinguishable from normal board textures.
3. Substantial intra-class variation in defect morphology.

These characteristics make this dataset particularly valuable for evaluating the robustness of deep learning-based inspection systems under realistic manufacturing conditions.

**Table 1:** Details of the investigated Peking university PCB dataset.

No.	Class name	No. images	No. defects	Training set	Validation set	Testing set
1	Missing holes (MH)	3664	7224	2932	366	366
2	Mouse Bites (MB)	3704	7368	2962	371	371
3	Open circuits (OC)	3480	7096	2784	348	348
4	Short circuits (SC)	3464	7016	2772	346	346
5	Spurs (S)	3504	7272	2802	351	351
6	Spurious coppers (SCo)	3520	7352	2816	352	352
Total	All 6 classes	21,336	43,328	17,068	2134	2134

#### Evaluation metrics and ablation study

To judge the performance of the presented YOLOv11-PCB approach for PCBs’ defect localization, we employed several evaluation metrics, including precision (P), FPS, average precision (AP), recall (R), mean average precision (mAP), and GFLOPs. All experimental results presented in Tables 1, 2, 3 represent original findings and were produced by our experiments, except where explicitly marked with reference citations. As indicated in Table 2, ablation research was carried out to assess each suggested module’s contribution.

The baseline model (Model 1) was YOLOv11. Subsequent models were incrementally enhanced:

- Model 2: Incorporated the EMA module into the backbone.
- Model 3: Integrated the CARAFE module into the Neck network.
- Model 4: Combined both EMA and CARAFE modules.
- Model 5: Further enhanced Model 4 by replacing the default IoU loss with the EIoU loss function to enhance small defect localization.

**Table 2:** Ablation experiment models.

Model	YOLOv11	EMA	CARAFE	EIoU
Model 1	√	×	×	×
Model 2	√	√	×	×
Model 3	√	×	√	×
Model 4	√	√	√	×
Model 5	√	√	√	√

Table 3 displays the outcomes of the ablation tests. The following are the main conclusions of the Ablation Study:

1. Impact of EMA Module (Model 2 vs. Model 1).

The introduction of EMA improved recall (R) by 0.2%, precision (P) by 0.1%, and mAP@0.5:0.95 by 1%, showing its effectiveness in recovering fine-grained features across different scales.

2. Effect of CARAFE Module (Model 3 vs. Model 1).

Integrating CARAFE increased mAP@0.5 by 0.3%, mAP@0.5:0.95 by 3.9%, and precision by 0.2%, indicating its ability to minimize feature information loss and enhance defect feature representation.

**Table 3:** Ablation experiment result of our approach. Significant values are bold.

	AP%						*P*%	*R*%	mAP@ 0.5%	mAP@ 0.5: 95%
	MH	MB	OC	SC	S	SCo				
Model 1	99.3	99.5	99.2	98.8	99.5	99.2	99.1	98.6	99	81
Model 2	99.4	99.3	99.4	98.8	99.3	99.2	99	98.8	99.1	82
Model 3	99.5	99.5	99.5	98.7	99.4	99.4	99.3	98.6	99.3	84.9
Model 4	99.5	99.5	99.4	99	99.5	99.5	99.5	98.8	99.4	87.2
Model 5	**99.5**	**99.5**	**99.5**	**99.5**	**99.5**	**99.5**	**99.7**	**99.8**	**99.5**	**90.7**

3. Combined Effect of EMA and CARAFE (Model 4).

The joint integration of EMA and CARAFE led to consistent improvements across all metrics, confirming their complementary roles in enhancing feature extraction and reassembly.

4. Superiority of EIoU Loss (Model 5).

Replacing the standard IoU loss with EIoU further refined localization accuracy, particularly for small defects. Model 5 achieved the highest performance with *P* = 99.7%, *R* = 99.8%, mAP@0.5 = 99.5%, and mAP@0.5:0.95 = 90.7%, surpassing the standard YOLOv11 by 0.6%, 1.2%, 0.5%, and 9.7%, respectively. A comparative analysis of different IoU-based loss functions (Table 4) revealed that EIoU outperformed alternatives such as Complete IoU (CIoU), Generalized IoU (GIoU), Superior IoU (SIoU), Distance IoU (DIoU), and Wise IoU (WIoU), achieving the highest precision (99.7%) and recall (99.8%). This confirms that EIoU is more effective in optimizing bounding box regression for PCB defect detection.

We evaluated various attention mechanisms integrated with YOLOv11, including CBAM, MLCA, CPCA, SE, RepVGG, SimAM, RepVGG + Carafe, and SimAM + Carafe (Table 5). The proposed model (incorporating EMA, CARAFE, and EIoU) consistently outperformed these variants, achieving the highest mAP@0.5:0.95 (90.7%), further validating its superior feature extraction and localization capabilities.

To assess the model’s competitiveness, we compared it with existing PCB defect detection approaches (Table 6), including:

- YOLO variants (YOLOv8, YOLOv9, YOLOv10, YOLOv11).
- Recently published models (CDI-YOLO, PCB-YOLO, TPH-YOLOv5, GCC-YOLO, Ghost-YOLOv8, etc.)

**Table 4:** Comparison of different losses on our investigated dataset.

Localization loss function	*P*%	*R*%	mAP@ 0.5%	mAP@ 0.5:95%
CIoU	99.3	98.6	99.4	87.2
GIoU	99.4	98.9	99.4	87.4
SIoU	99.5	98.7	99.4	86.5
DIoU	99.4	98.9	99.4	87.4
WIoU	99.6	98.9	99.5	88
EIoU	**99.7**	**99.8**	**99.5**	**90.7**

**Table 5:** Comparison of metrics from several attention mechanism models combined with YOLOv11.

Models	MH	MB	OC	SC	S	SCo	*P*%	*R*%	mAP@ 0.5%	mAP@ 0.5:95%
YOLO11 + CBAM	99	99.3	99	98.4	99.4	98.7	98.1	97.7	99	76
YOLO11 + MLCA	99.2	99	98.9	98.5	99	98.9	97.9	97.6	98.9	73.8
YOLO11 + CPCA	99.4	99.5	99.4	98.8	99.5	99.4	98.8	98.6	99.3	81.4
YOLO11 + SE	99.4	98.9	99	98.4	99.4	98.9	98.2	97.7	99	75.7
YOLO11_repvgg	99.5	99.5	99.5	98.6	99.5	99.5	99.4	98.6	99.3	87.1
YOLO11 + simAM	99.5	99.5	99.3	99.1	99.5	99.5	99.4	99	99.4	89.1
YOLO11_repvgg_Carafe	99.5	99.5	99.4	98.9	99.5	99.4	99.3	98.7	99.4	85.5
YOLO11_simAm_carafe	99.5	99.5	99.2	98.9	99.4	99.4	99.5	98.7	99.3	86.1
The proposed	99.5	99.5	99.5	99.5	99.5	99.5	99.7	99.8	99.5	90.7

**Fig. 6:**
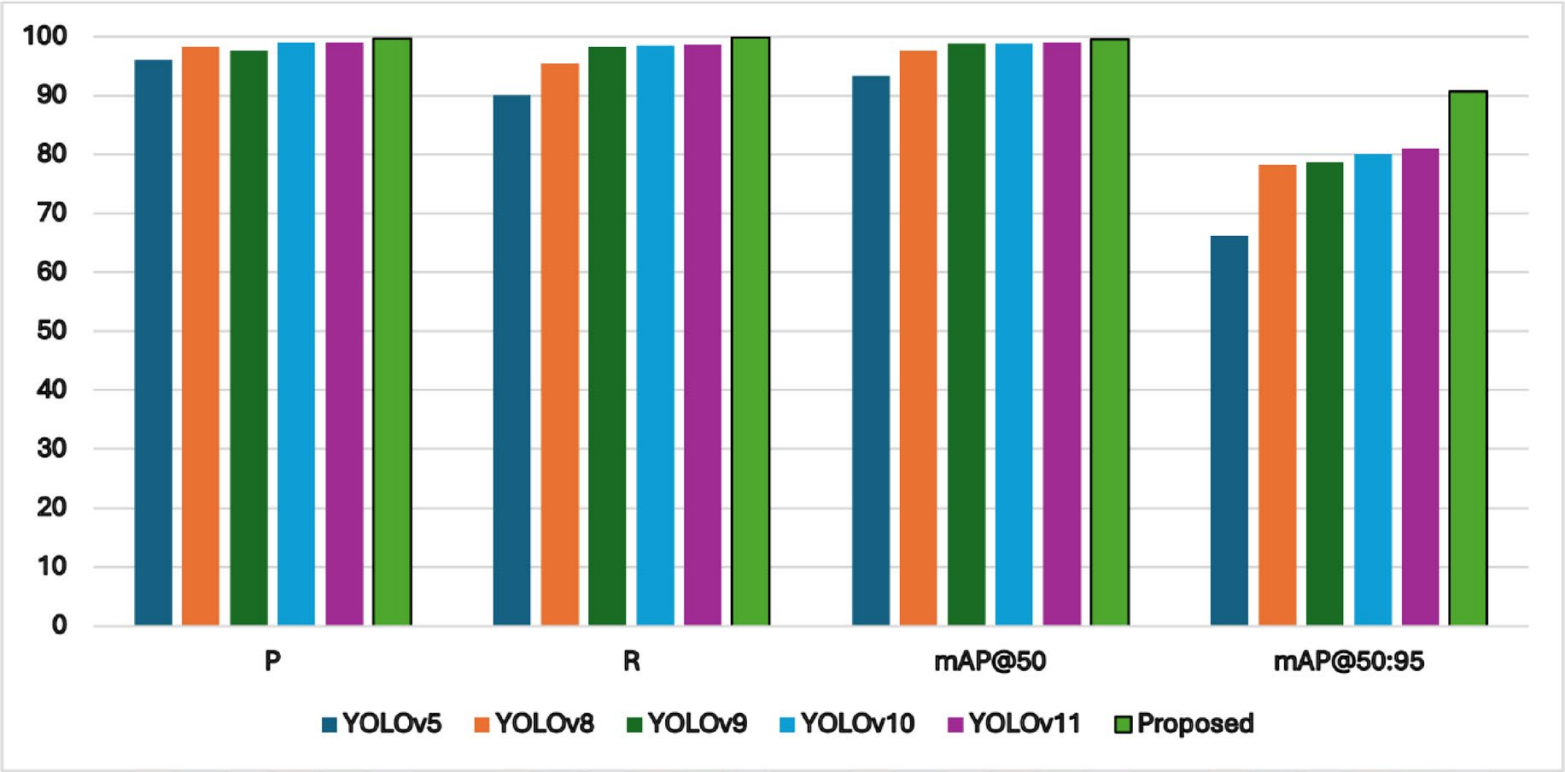
Comparison of our model and the advanced YOLO series models.

**Fig. 7:**
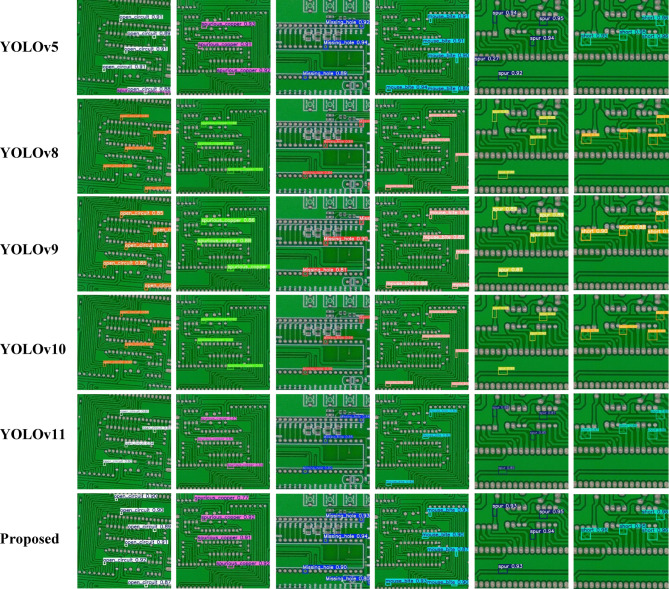
Localization results of advanced object detection models.

**Fig. 8:**
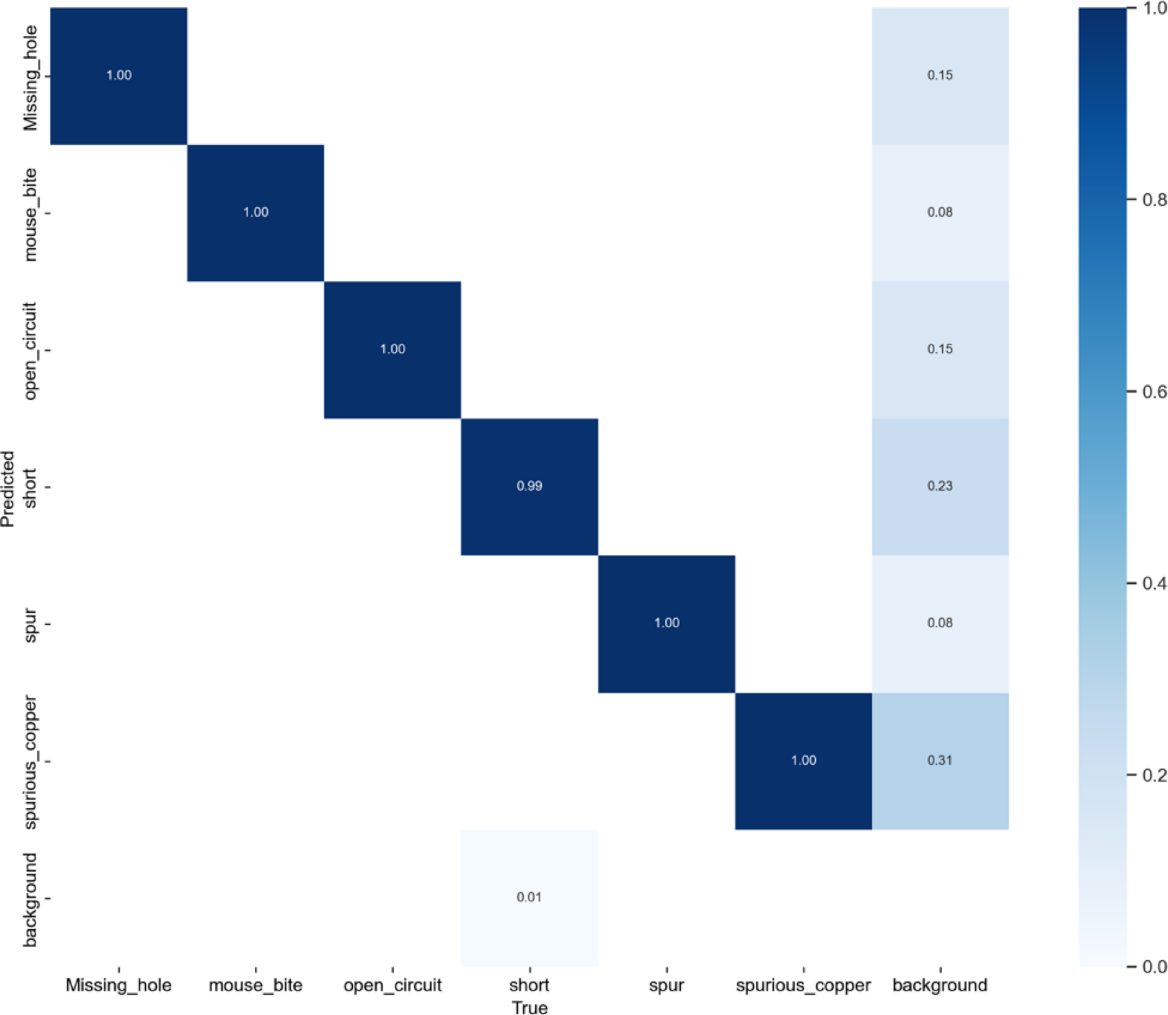
The confusion matrix of the proposed YOLOv11-PCB model.

Our model established superior performance in both detection accuracy (mAP@0.5 = 99.5%) and generalization capability (mAP@0.5:0.95 = 90.7%), significantly outperforming existing methods. Notably, it achieved higher recall (99.8%) than all compared models, indicating robust defect localization even for challenging cases. The comparative analysis of YOLOv11-PCB with leading YOLO-based architectures, including YOLOv5, YOLOv8, YOLOv9, YOLOv10, and YOLOv11, is presented in Table 6; Fig. 6, with detection outcomes visualized in Fig. 7. Experimental results demonstrate that YOLOv11-PCB surpasses existing small-object detection models on the investigated PCB dataset, achieving superior localization accuracy across all defect categories. This improvement highlights the model’s robustness in identifying minute targets, a critical requirement for PCB inspection. Figure 7 further validates the model’s efficacy, displaying precise defect localization even in densely structured PCB layouts. The visual evidence corroborates the model’s ability to maintain high detection precision under challenging conditions. As quantified in Table 7, the proposed model achieves an industry-leading inference speed of 227.2 FPS, outperforming all compared methods. While Ghost-YOLOv8 attains the lowest computational load (GFLOPs), YOLOv11-PCB strikes an optimal balance, delivering real-time performance without compromising detection accuracy. At 22.6 GFLOPs, YOLOv11-PCB strikes a balance between Ghost-YOLOv8’s efficiency (9.2 GFLOPs) and heavy models like YOLOv9 (103 GFLOPs). However, its 8GB GPU memory requirement may constrain edge deployment—quantization to INT8 could reduce this by 4x with minimal accuracy loss, a key direction for industrial adoption. Figure 8 displays the confusion matrix for the testing dataset of the suggested YOLOv11-PCB model. The 99% accuracy for short circuits (vs. 99.83% overall) indicates residual challenges with low-contrast conductive bridges. Qualitative inspection reveals these errors occur primarily in dense via regions, where background clutter mimics defect signatures.

**Table 6:** A comparison of the suggested model’s metrics with those of other published models.

	AP%						*P*%	*R*%	mAP@0.5%	mAP@0.5:95%
	MH	MB	OC	SC	S	SCo				
CDI-YOLO^48^	98.9	97.8	98.3	97.6	98.3	98.6	97.1	96.4	98.3	51.1
Improves YOLOv5s^17^	99.3	98.89	98.62	98.06	98.54	99.2	98.8	97.2	98.77	68.4
PCB-YOLO^13^	99.5	99	98.6	99.4	96.2	95.9	99	96.7	98.1	64.2
TPH-YOLOv5^45^	99.5	98.3	98.4	99	95.9	95.1	97.9	96.8	97.7	66.6
Light-YOLOv5^16^	98.8	93.8	94.1	95.4	86.9	91.1	96	89.9	93.3	54.8
Improved YOLOv7^8^	99.5	97	90.2	99	91.6	93	95.1	91.1	92.8	55
YOLO‑FGD^4^	99.5	98.4	98.1	98.6	99.5	99.2	98	98.9	98.8	57.2
GCC-YOLO^43^	99.5	98.3	98.6	99.2	97.3	96.1	99	97.3	98.2	76.7
Ghost-YOLOv8^2^	98.9	98.7	98.9	99.2	97.6	99.7	98.4	98.7	99.4	77.2
DsP-YOLO^44^	99.5	92.6	94.3	99.5	93.4	95.2	94.7	94.3	95.8	-
YOLOv8^43^	99.5	98.2	95.8	98.7	94.6	98.8	98.2	95.4	97.6	78.3
YOLOv9^4^	99.4	99.3	98.5	98.8	99.3	99	97.7	98.2	98.5	78.7
YOLOv10	99.4	99.5	99.1	98.6	99.5	99.3	99.1	98.5	98.8	80
YOLOv11	99.3	99.5	99.2	98.8	99.5	99.2	99.1	98.6	99	81
The proposed	**99.5**	**99.5**	**99.5**	**99.5**	**99.5**	**99.5**	**99.7**	**99.8**	**99.5**	**90.7**

**Table 7:** Comparison of our model’s inference time and GFLOPs^*^ with those of other models.

Method	GFLOPs	Frame/sec (FPS)
YOLOv3-spp^4^	283.8	59.8
Yolov5^43^	15.8	89.3
Improved YOLOv5s^17^	16.42	41
YOLOv7^8^	190.6	46.62
YOLOv7-tiny^4^	13.9	84
YOLOv7 + Triplet + RFE + WIoU^8^	210.3	49.02
Yolov8^10^	28.4	79.4
Ghost-YOLOv8^2^	**9.2**	125
YOLOv9c^4^	103.2	54.6
RDD-YOLO^44^	145.6	43.8
YOLOR^48^	114.1	58
YOLO-MBBI^6^	12.8	48.9
YOLO-FGD model^4^	11.5	104.8
CDI-YOLO^48^	12.6	128
GCC-YOLO^43^	17.8	68
DsP-YOLO^44^	28.5	81.3
AKPLNet^40^	312.5	6.3
Faster R-CNN with SF-PSPyramid^35^	124.78	30
YOLOv5 + FPN + modified CIoU^36^	20	90
YOLOv10	24.5	217.39
YOLOv11	21.3	222.2
The proposed (YOLOv11 + EMA + CARAFE + EIoU)	22.6	**227.2**

^*^GFLOPs: Giga Floating Point Operations per Second.

The experimental results prove that the proposed enhancements—EMA for multi-scale feature refinement, CARAFE for improved feature reassembly, and EIoU for precise localization—collectively contribute to a highly efficient and accurate PCB defect detection system. The model achieves state-of-the-art performance while maintaining computational efficiency, making it suitable for real-world industrial applications. This work provides a robust framework for automated PCB inspection, with potential extensions to other defect detection tasks in electronics manufacturing.

While YOLOv11-PCB demonstrates superior performance in defect detection, several limitations remain. First, the model’s reliance on high-resolution imagery and GPU acceleration may limit deployment on low-power edge devices. Second, although the dataset was augmented for diversity, it may not fully capture the variability of real-world PCB designs across different manufacturers. Third, the model’s performance in detecting overlapping or occluded defects in highly cluttered environments warrants further investigation. Future work will explore model quantization, domain adaptation techniques, and integration with real-time AOI systems to enhance deployment feasibility in diverse industrial settings.

### Experimental results for deep-PCB database

The performance of the proposed YOLOv11-PCB model was further evaluated using the DeepPCB dataset, which comprises 3,600 annotated black and white images labeled with six common PCB defect types: open circuits, short circuits, mouse bites, spurs, pinholes, and spurious copper^34^. Captured using a linear scan CCD in real-world environments, the dataset ensures practical relevance. To maintain evaluation integrity, it was systematically divided into training (80%), validation (10%), and testing (10%) subsets with no overlap.

**Fig. 9:**
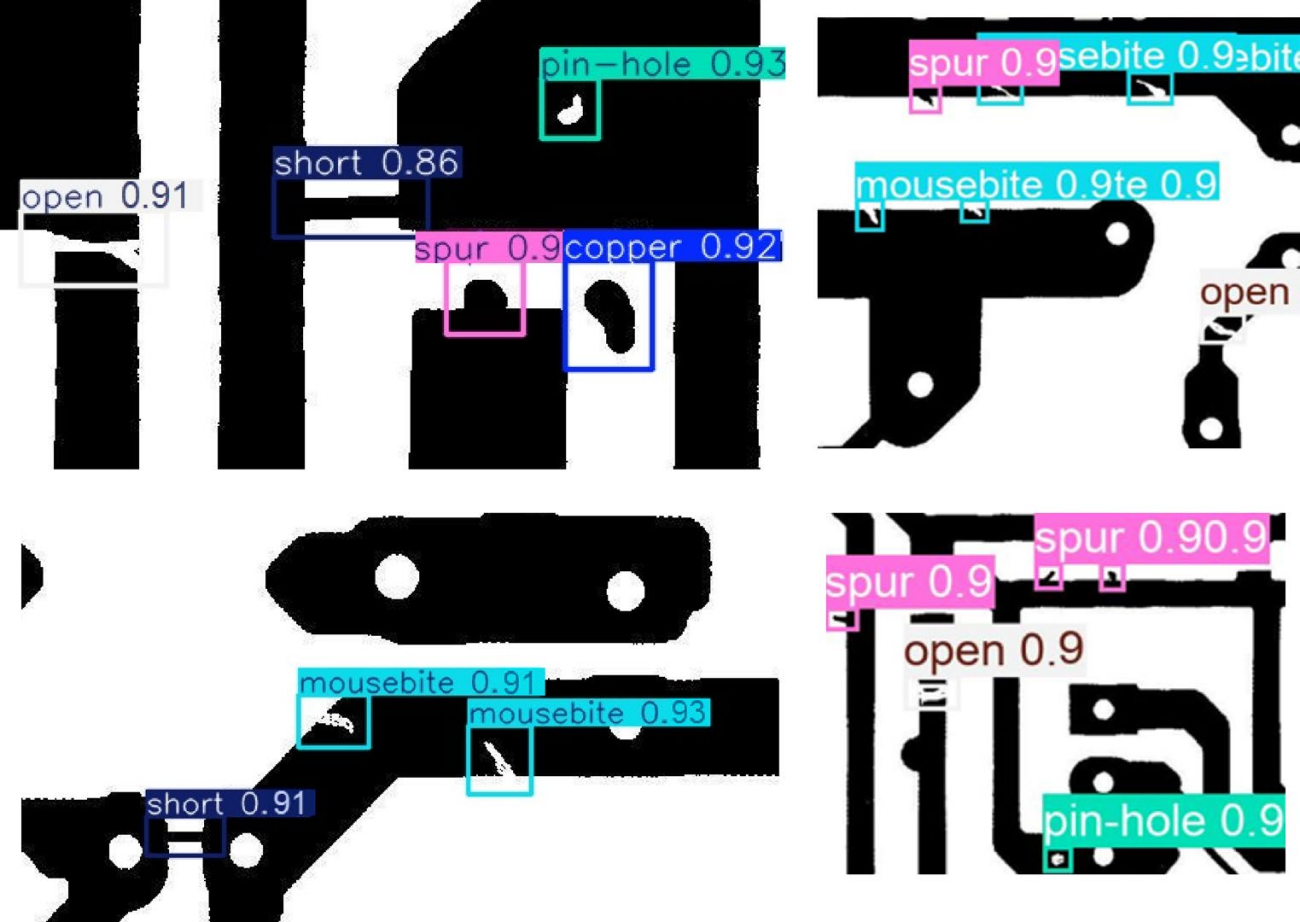
Test results of DeepPCB, showing detection outcomes across six defect categories.

Despite the challenges posed by the dataset’s small, low-resolution black and white images, YOLOv11-PCB demonstrated robust detection capabilities. As illustrated in Fig. 9, the model achieved high accuracy across all defect categories, confirming its effectiveness under constrained visual conditions.

For benchmarking, YOLOv11-PCB was compared against ten state-of-the-art models, including MobileNetV3, Faster R-CNN, ShuffleNetV2-YOLOv5, Ghost-YOLOv8, and several YOLO variants (YOLOX, YOLOv5, YOLOv7, YOLOv8, YOLOv10, and YOLOv11). As shown in Table 8, YOLOv11-PCB outperformed all competitors, achieving the highest precision (98.4%), recall (96.5%), mAP@0.5% (98.9%), and mAP@0.5:0.95% (81%). These results highlight its superior feature extraction and defect localization performance, particularly in detecting all defect types with exceptional accuracy.

Despite the high accuracy observed across multiple models on the DeepPCB dataset, this benchmark remains particularly challenging due to its low-resolution grayscale imagery, minimal contrast, and subtle defect variations—conditions that closely resemble real-world industrial environments. In this context, even marginal improvements in detection precision (e.g., 0.5% to 1% in mAP) can significantly reduce false negatives and enhance production yield. While mAP@0.5 values are closely clustered, our model achieves superior localization precision with an mAP@0.5:0.95 of 81% (compared to 79.2% for YOLOv11 and 75.8% for YOLOv10), the highest recall (96.5%), and real-time inference at 227.2 FPS—demonstrating its robustness and practical relevance under constrained visual conditions.

**Table 8:** Comparative performance metrics of the proposed YOLOv11-PCB model and other state-of-the-art models on the DeepPCB dataset.

Model	AP%						*P*%	*R*%	mAP@0.5%	mAP@0.5:95%
	Pin-hole	Mousebite	Open	Short	Spur	Copper				
ShuffleNetV2-YOLOv5^18^	82	61.8	70.9	35.7	20.8	27	54.5	53.4	49.7	-
Faster RCNN^2^	96.5	98.4	97.2	78.7	89.1	76.4	-	83.5	87.8	-
Ghost-YOLOv8^2^	96.2	96.1	96.6	76.8	74.3	78.6	-	82.5	87	-
YOLOX^40^	97.6	96.2	95.3	95.5	97.2	96.3	-	-	96.3	-
MobileNetV3^18^	77.9	47.5	66.9	42.8	15.2	15.3	47	50.6	44.3	-
YOLOv5^2^	97.1	96.6	95.5	77.3	74.5	78.7	85.2	81.5	86.3	-
YOLOv7^2^	96.1	97.4	95.6	86.3	80	83.2	-	81.2	82.3	-
YOLOv8^2^	96.3	96	96.4	77.1	73.9	77.5	-	83.8	86.2	-
YOLOv10	98.9	98.3	98.4	96.1	98.1	99.1	97	96.2	98.2	75.8
YOLOv11	99.4	98.2	98.7	96.9	98.2	99.4	97.3	95.7	98.5	79.2
The proposed	99.5	98.7	99.1	97.8	99.2	99.5	98.4	96.5	98.9	81

Statistical analysis using multiple evaluation metrics was conducted to uncover data patterns and explain variations in experimental outcomes. Key statistical indicators—including mean, standard deviation (SD), and variance—were calculated for all performance metrics. Table 9 summarizes the performance and statistical analysis of the proposed YOLOv11-PCB model on both the DeepPCB and Peking University datasets.

The results indicate that YOLOv11-PCB consistently achieves high detection accuracy, with performance improving as training epochs increase. However, for the Peking University dataset, accuracy plateaus between 300 and 500 epochs, suggesting convergence and model stability. Comparative analysis further reveals that YOLOv11-PCB outperforms competing methods by achieving higher mean values across all metrics.

Notably, the model demonstrates superior performance on the PKU-market-PCB dataset, as evidenced by lower standard deviation and variance values, confirming its robustness and consistency. Table 10 provides a comprehensive statistical comparison between YOLOv11-PCB and other state-of-the-art models across both datasets, further validating the effectiveness of the proposed approach.

**Table 9:** Performance evaluation of the proposed YOLOv11-PCB model across two PCB datasets: Peking university and DeepPCB.

	Peking University PCB dataset					DeepPCB PCB dataset				
	Accuracy %	*P* %	*R* %	F1-Score %	mAP@ 0.5%	Accuracy %	*P* %	*R* %	F1-Score %	mAP@ 0.5%
Ours at Epoch = 100	97.5	97.5	96.8	97.15	98.1	95.33	95.6	93.5	94.54	97.1
Ours at Epoch = 200	98.33	98.1	98.1	98.10	99.1	95.67	96.5	94.1	95.28	97.2
Ours at Epoch = 300	99.83	99.7	99.8	99.75	99.5	96.3	96.9	95	95.94	97.7
Ours at Epoch = 400	99.83	99.8	99.7	99.75	99.5	96.5	97.2	95.3	96.24	98.1
Ours at Epoch = 500	99.83	99.7	99.8	99.75	99.5	97.3	98.4	96.5	97.44	98.9
Mean	99.03	98.96	98.84	98.90	99.14	96.22	96.92	94.88	95.89	97.80
SD	0.95	0.97	1.21	1.08	0.54	0.68	0.92	1.03	0.97	0.66
Variance	0.90	0.93	1.46	1.17	0.29	0.47	0.84	1.07	0.94	0.43

## Conclusion

This paper introduces YOLOv11-PCB, a high-performance deep learning framework tailored for precise and real-time defect-detection in PCBs. The proposed model integrates three key innovations: the EMA module for adaptive feature extraction, the CARAFE mechanism for enhanced spatial resolution, and the EIoU loss function for improved bounding box regression. Together, these components address critical challenges in PCB inspection, including the detection of minute defects, the need for real-time inference, and the constraints of industrial deployment. To validate the model’s effectiveness and generalizability, extensive experiments were conducted on two benchmark datasets. On the Peking University PCB dataset, which includes 21,336 high-resolution color images, YOLOv11-PCB achieved a mAP@0.5% of 99.5% and a mAP@0.5:0.95% of 90.7%. On the DeepPCB dataset, consisting of 3,600 grayscale images captured in real-world industrial settings, the model attained a mAP@0.5% of 98.9% and a mAP@0.5:0.95% of 81%. These results significantly outperform state-of-the-art models, including YOLOv8, YOLOv9, and YOLOv10, while maintaining an industry-leading inference speed of 227.2 FPS. Ablation studies further confirm the individual contributions of each module, with the EIoU loss alone improving mAP@0.5:0.95% by 9.7% over the baseline YOLOv11. These findings underscore the robustness, scalability, and efficiency of the proposed architecture, making it a strong candidate for integration into AOI systems on production lines. The proposed architecture bridges the gap between academic innovation and industrial application, offering a practical solution for high-speed, high-accuracy PCB inspection. EMA, CARAFE, and EIoU are well established, but their integration into a unified, efficient architecture for PCB defect detection is a novel contribution. Its modular design also allows for easy adaptation to other industrial defect detection tasks, paving the way for broader adoption in smart manufacturing environments. Despite its strong performance, the model’s reliance on high-resolution input and GPU acceleration may limit deployment on low-power edge devices. Future work will aim to optimize the model for edge deployment through quantization and pruning, improve domain adaptability to accommodate diverse PCB designs, and validate performance to ensure generalizability across datasets from various manufacturers and production environments.

**Table 10:** Comparative statistical analysis of various models on the Peking university and DeepPCB datasets.

Dataset	Model	Accuracy %			P %			F1-score %		
		Mean	SD	Variance	Mean	SD	Variance	Mean	SD	Variance
Peking University PCB	RCNN^47^	92.02	0.32	0.09	92.91	1.23	1.52	92.93	0.9	0.82
	Faster RCNN^2^	92.73	0.28	0.08	92.49	0.62	0.39	92.72	0.56	0.31
	YOLOv5^47^	92.39	0.58	0.34	92.67	0.84	0.71	92.67	0.66	0.44
	MuSAP-YOLOv5^47^	93.79	0.59	0.35	94.07	0.48	0.23	94.17	0.48	0.23
	Ours	99.03	0.95	0.9	98.84	0.97	0.93	98.9	1.08	1.17
DeepPCB PCB	RCNN^47^	92.95	0.57	0.32	92.41	0.71	0	92.71	0.56	0.31
	Faster RCNN^2^	93.22	0.9	0.81	92.79	0.76	0.7	92.64	0.52	0.27
	YOLOv5^47^	92.86	0.5	0.25	93.39	0.8	0.34	92.94	0.4	0.16
	MuSAP-YOLOv5^47^	94.2	0.75	0.56	94.32	0.73	0.54	94.32	0.84	0.70
	Ours	96.22	0.68	0.47	96.92	0.92	0.84	95.89	0.97	0.94

## Data Availability

The datasets analyzed during the current study are available at https://www.kaggle.com/datasets/norbertelter/pcb-defect-dataset, https://universe.roboflow.com/tack-hwa-wong-zak5u/deeppcb-4dhir/dataset/5.
